# Psychological responses and associated factors during the initial stage of the coronavirus disease (COVID-19) epidemic among the adult population in Poland - a cross-sectional study

**DOI:** 10.1186/s12889-021-11962-8

**Published:** 2021-10-23

**Authors:** Bernard Sozański, Agnieszka Ćwirlej-Sozańska, Agnieszka Wiśniowska-Szurlej, Krystian Jurek, Patryk Górniak, Karol Górski, Anna Englert-Bator, Lidia Perenc

**Affiliations:** 1 Institute of Medicine, Medical College of Rzeszow University, Rzeszów, Poland; 2 Polish Society of Public Health, Branch of Rzeszow, Rzeszow, Poland; 3 Gerontoprophylaxis Laboratory, Centre for Innovative Research in Medical and Natural Sciences, Rzeszów University, Rzeszów, Poland; 4 Institute of Health Sciences, Medical College of Rzeszow University, Rzeszów, Poland; 5 Scientific Club of Physiotherapy in Geriatrics and Health Prevention, Section of Physioprophylaxis, Institute of Health Sciences, Medical College of Rzeszow University, Rzeszów, Poland; 6 Institute of Pedagogy, College of Social Sciences of Rzeszow University, Rzeszów, Poland

**Keywords:** SARS-CoV-2, stress, depression, anxiety, quality of life

## Abstract

**Introduction:**

The study aimed to assess the emotional state, the occurrence of symptoms of depression, anxiety, and stress, as well as the quality of life of adults living in Poland during the first weeks of the COVID-19 pandemic.

**Method:**

The study was conducted on a group of 700 people aged 18 and over living in Poland. An anonymous online questionnaire was used in this cross-sectional study. The psychological impact of COVID-19 was measured using the Revised Event Impact Scale (IES-R) and the Depression, Anxiety, and Stress Scale (DASS - 21). The quality of life was assessed using the WHOQOL-BREF.

**Results:**

In Poland, a high average level of post-traumatic stress was found as a result of the COVID-19 pandemic, with at least the minimum level occurring in all surveyed people. There was also a high incidence of depression (48.00%), anxiety (39.29%), and stress (54.86) in the first phase of the pandemic. The average level of quality of life in Poland was the lowest for the physical domain and amounted to 49.56 (SD = 11.71). The standard of living in the psychological domain was 60.26 (SD = 13.14).

**Conclusions:**

The pandemic is having a significant impact on human mental health. The very high average levels of post-traumatic stress, stress, anxiety, and depression as well as low quality of life make it necessary to consider interventions that will favor the use of more adaptive defense mechanisms and build mental resilience during an infectious disease pandemic and its long-term consequences.

## Background

The disease caused by the SARS-CoV-2 (COVID-19) virus infection was first diagnosed in December 2019 in Wuhan, China, spreading to other countries of the world [1]. On January 30, 2020, the World Health Organization (WHO) announced the outbreak of the COVID-19 epidemic [2]. High infectivity and the rapid spread of the virus have become a particular problem [3, 4]. In Poland, the first case of COVID-19 infection was detected on March 4, 2020. From that moment on, there was a gradual development of the pandemic in the country. On March 11, the Minister of National Education in Poland issued a regulation on the temporary limitation of the functioning of education system units [5]. Further restrictive restrains were introduced very quickly. At the beginning of the pandemic, the problem of the lack of masks, medical equipment and disinfectants appeared in Poland [6]. From March to July 2020, over 38,000 cases of infection and over 1,562 deaths were found in Poland [7].

The transmissibility of COVID-19 has been estimated at 4.08 [8, 9]. It is estimated that the average incubation period for COVID-19 is 5.2 days [10]. The course of the disease is very diverse [11–14]. The mortality risk among confirmed cases is estimated at 3–6% [15]. The isolation of a large number of people in Poland in March affected many aspects of their lives. The COVID-19 epidemic has caused serious threats to people's physical health and lives. Society needs a quick understanding of mental health condition during a pandemic [16]. Research on the impact of health and life threatening situations and isolation on mental health is particularly important in the context of the uncertainty surrounding the outbreak of an epidemic on such an unprecedented scale.

Past studies in areas affected by epidemics have confirmed that the introduced restrictions and isolation negatively affect the emotions felt by individuals [17]. During the outbreak of acute respiratory syndrome (SARS) epidemic, many studies assessed the psychological impact on the community, revealing the activation of many previously unrecognized disorders and mental problems [18]. A high level of fear of illness and death, as well as a feeling of helplessness and stigmatization were found [19]. Elderly, female, more educated people, with a higher risk of perceiving SARS, with a moderate level of anxiety, with a positive contact history, and people with symptoms similar to SARS, were more likely to take preventive measures against infection [20]. Isolation from other members of the community, combined with other risk factors during an epidemic (SARS), increased the likelihood of developing depressive symptoms in the future and a 2-3-fold increase in the symptoms of post-traumatic stress disorder (PTSD) [21–23]. Additionally, during a pandemic, isolation weakens important ways to combat stress, such as physical activity and spiritual coping, increasing mental and physical stress [24].

### Objective

To the best of our knowledge, this research is one of the first studies in Poland to assess the level of anxiety, stress, depression and the quality of life of Poles during the first weeks of the COVID-19 epidemic. The aim of the study was to assess the emotional state, the occurrence of symptoms of depression, anxiety and stress, as well as the quality of life of adults living in Poland during the first weeks of the COVID-19 pandemic in Poland. Health behavior was also analyzed and the impact of sociodemographic and health factors on the psychophysical condition of Poles during the COVID-19 pandemic was assessed.

## Methods

### Setting and participants

An anonymous online questionnaire was used in this cross-sectional study. A snowball sampling strategy, focused on recruiting the general public living in Poland during the epidemic of COVID-19, was utilized. The online questionnaire in Polish was posted on various websites and sent to various universities and institutions in Poland in order to reach the widest possible group of respondents, with a request to further disseminate the study. The research methodology was largely based on the methodology developed by the research team of Wang et al. [25], thanks to which it is possible to compare the psychological situation in our countries. The study was conducted on a group of 823 people aged 18 and over living in Poland. The inclusion criteria for the study were: age 18 and over and informed consent to participate in the study. Finally, 700 complete survey questionnaires were used for the analysis. Data have been collected in the period from April 1 to May 30, 2020.

The calculation of the sample size was based on the following assumptions: a 95% (0.95) confidence level and a fraction size of 0.5 with a maximum estimation error of 4%.

The study design was approved by the Bioethics Committee of the University of Rzeszów (Resolution No. 1/04/2020). In accordance with Declaration of Helsinki, the subjects were provided with information about the aim and the course of the study, and expressed their informed consent to participate. The respondents also were informed about the possibility of withdrawing from the study at any stage.

The manuscript was prepared in accordance with the STROBE guidelines.

### Procedure

Survey respondents completed questionnaires in Polish via a Google Form. All respondents gave their informed consent to participate in the study.

### Survey development

The survey questionnaire was prepared based on an analysis of studies assessing the psychological impact of other viral epidemics [26, 27], as well as Chinese studies of the COVID-19 epidemic [25, 28]. The structured questionnaire consisted of questions that covered several areas: (1) sociodemographic data; (2) general health; (3) current health condition; (4) questions about the coronavirus (COVID-19); (5) prophylaxis; (6) additional information required in relation to COVID-19; (7) psychological effects of the COVID-19 outbreak; (8) mental health status, (9) quality of life assessment.

The psychological impact of COVID-19 was measured using the Revised Event Impact Scale (IES-R) [29, 30] and the Depression, Anxiety and Stress Scale (DASS - 21) [31–34]. The quality of life of the respondents during the pandemic was also assessed using the WHOQOL-BREF - a short version of the Quality of Life Questionnaire - QoL [35].

### Statistical methods

The collected data were analyzed using TIBCO Software Inc. (2017) Statistica (data analysis software system), version 13.

The quantitative characteristics of the studied population were presented in terms of the analyzed variables using the mean ± standard deviation (SD) for measurable variables and the results of the IES-R and DASS subscales as well as the WHOQOL-BREF or the number of subjects (in per cent) for categorical variables, respectively.

Linear regression models were used in order to assess the impact of individual variables such as sociodemographic variables, variables describing physical symptoms, health services, contact history, as well as knowledge and concerns on the IES-S score, WHOQOL-BREF scores and the values of the DASSL subscales.

P-values less than 0.05 were considered statistically significant.

## Results

### General assessment of the psychological impact and quality of life during the initial phase of the pandemic in Poland

The psychological impact of the COVID-19 outbreak, as measured by the IES-R scale, showed an average level of 29.75 (SD = 16.05). Of all respondents, 271 (38.71%) reported minimal psychological impact, 145 (20.71%) - mild psychological impact, 51 (7.29%) - moderate (scores 33-36), and strong - 233 (33.29%).

The mean level of depression in the DASS-21 depression subscale was 9.85 (SD = 7.77). No depression was found in 364 subjects (52.00%), mild depression was found in 103 subjects (14.71%), moderate depression in 154 (22.00%), and severe and extremely severe in 48 (6.86%) and 31 (4.43%), respectively. The mean level of anxiety in the DASS-21 anxiety subscale was 6.70 (SD = 7.01). No anxiety was found in 425 (60.71%) persons, mild anxiety was assessed in 50 (7.14%), moderate in 146 (20.86%), and strong and extremely severe anxiety in 27 (3.86%) and 52 (7.43%) subjects, respectively.

The mean level of stress in the stress subscale of the DASS-21 scale was 11.68 (SD = 8.18). The increased level of stress was not found in 316 (45.14%) subjects, mild stress occurred in 261 (37.29%) people, moderate in 83 (11.86%), and strong in 40 (25.71%).

The average level of quality of life in Poland was the lowest for the physical domain and amounted to 49.56 (SD = 11.71). The highest average standard of living was found in the environmental domain (mean = 65.83; SD = 15.12) and the social domain (mean = 65.82; SD = 19.41). The standard of living in the psychological domain was 60.26 (SD = 13.14).

### Socio-demographic variables and psychological impact and quality of life

The sociodemographic characteristics are presented in Table 1. Most of the respondents were women (59.57%), people living in a relationship (67.00), with higher education (55.43%), professionally active (58.43%), childless (54.57%), living in households with complex out of 4 people (31.00%) whose financial resources were sufficient to meet their needs to a moderate extent (31.57%). The mean age of the respondents was 36.5 (SD = 15.6). Female gender was significantly associated with higher scores on the DASS anxiety subscale (B = 0.15, 95% CI: 0.08 to 0.23).

**Table 1:** Association between demographic variables and the psychological impact of the 2019 coronavirus disease (COVID-19) outbreak as well as adverse mental health status during the epidemic (*n* = 700)

Variables	***N (%)*** ***Mean (SD)***	Impact of event		Stress		Anxiety		Depression		WHOQOL	
		Beta (95% Confidence Interval) B (95% CI)	R-Squared (R^**2**^)	Beta (95% Confidence Interval) B (95% CI)	R-Squared (R^**2**^)	Beta (95% Confidence Interval) B (95% CI)	R-Squared (R^**2**^)	Beta (95% Confidence Interval) B (95% CI)	R-Squared (R^**2**^)	Beta (95% Confidence Interval) B (95% CI)	R-Squared (R^**2**^)
**Gender**											
Female	417 (59,57)	-0,06	0,003	0,02	0,000	0,15***	0,024	0,05	0,003	-0,01	0,000
		(-0,13 to 0,02)		(-0,06 to 0,09)		(0,08 to 0,23)		(-0,02 to 0,13)		(-0,09 to 0,06)	
Male	283 (40,43)	Reference		Reference		Reference		Reference		Reference	
**Age** (Years)	36,5	0,12**	0,014	0,08**	0,006	0,11**	0,013	0,04	0,002	-0,05	0,003
	(15,6)	(0,05 to 0,19)		(0,00 to 0,15)		(0,04 to 0,19)		(-0,03 to 0,12)		(-0,13 to 0,02)	
**Marital status**											
Single	231 (33,00)	-0,08*	0,007	-0,05	0,003	-0,03	0,001	0,07	0,005	-0,10*	0,009
		(-0,15 to -0,01)		(-0,13 to 0,02)		(-0,11 to 0,04)		(0,00 to 0,15)		(-0,17 to -0,02)	
In relationship	469 (67,00)	Reference		Reference		Reference		Reference		Reference	
**Educational attainment**											
Vocational at best	65 (9,29)	0,21***	0,024	0,25***	0,032	0,31***	0,044	0,24***	0,037	-0,25***	0,052
		(0,09 to 0,32)		(0,14 to 0,36)		(0,20 to 0,42)		(0,13 to 0,35)		(-0,36 to -0,14)	
Secondary	247 (35,29)	-0,08		-0,12*		-0,19***		-0,07		0,03	
		(-0,19 to 0,03)		(-0,23 to -0,01)		(-0,30 to -0,08)		(-0,18 to 0,04)		(-0,08 to 0,14)	
Higher	388 (55,43)	Reference		Reference		Reference		Reference		Reference	
**Employment status**											
Pupil/student	189 (27,00)	-0,08*	0,020	-0,07	0,009	-0,11**	0,018	-0,01	0,022	0,04	0,013
		(-0,15 to 0,00)		(-0,14 to 0,01)		(-0,19 to -0,04)		(-0,08 to 0,07)		(-0,03 to 0,12)	
Professionally active	409 (58,43)	-0,10**		-0,06		-0,06		-0,15***		0,10*	
		(-0,18 to -0,03)		(-0,13 to 0,02)		(-0,14 to 0,02)		(-0,22 to -0,07)		(0,02 to 0,17)	
Profesionally inactive	102 (14,57)	Reference		Reference		Reference		Reference		Reference	
**Status as a parent**											
Has children under 16	178 (25,43)	-0,07	0,026	-0,02	0,010	-0,05	0,019	-0,14**	0,013	0,13**	0,015
		(-0,17 to 0,03)		(-0,12 to 0,08)		(-0,15 to 0,05)		(-0,24 to -0,05)		(0,04 to 0,23)	
Has children aged 16 and over	140 (20,00)	0,20***		0,11*		0,17***		0,13**		-0,12*	
		(0,10 to 0,30)		(0,01 to 0,21)		(0,07 to 0,27)		(0,03 to 0,23)		(-0,22 to -0,02)	
No children	382 (54,57)	Reference		Reference		Reference		Reference		Reference	
**Household size**											
One person	49 (7,00)	0,01	0,003	-0,02	0,002	0,01	0,003	0,03	0,001	-0,04	0,001
		(-0,11 to 0,14)		(-0,14 to 0,1)		(-0,11 to 0,13)		(-0,09 to 0,16)		(-0,16 to 0,08)	
Two people	124 (17,71)	-0,07		-0,05		0,00		-0,01		0,04	
		(-0,17 to 0,03)		(-0,15 to 0,05)		(-0,1 to 0,10)		(-0,11 to 0,09)		(-0,06 to 0,14)	
Three people	149 (21,29)	0,04		0,05		0,05		0,00		-0,02	
		(-0,06 to 0,14)		(-0,05 to 0,15)		(-0,05 to 0,15)		(-0,10 to 0,10)		(-0,12 to 0,08)	
Four people	217 (31,00)	-0,01		0,01		-0,01		-0,04		0,01	
		(-0,11 to 0,09)		(-0,08 to 0,11)		(-0,10 to 0,09)		(-0,14 to 0,06)		(-0,09 to 0,11)	
Five people or more	161 (23,00)	Reference		Reference		Reference		Reference		Reference	
**Financial resources to meet the needs**											
Are not enough	43 (6,14)	0,05	0,035	0,06	0,034	0,04	0,052	0,13	0,049	-0,29***	0,128
		(-0,08 to 0,18)		(-0,07 to 0,19)		(-0,09 to 0,16)		(0,00 to 0,26)		(-0,41 to -0,16)	
In small extent	57 (8,14)	0,20**		0,18**		0,18**		0,20***		-0,28***	
		(0,08 to 0,32)		(0,06 to 0,30)		(0,06 to 0,30)		(0,09 to 0,32)		(-0,39 to -0,16)	
Moderate	221 (31,57)	0,00		0,02		0,08		0,00		0,07	
		(-0,10 to 0,10)		(-0,09 to 0,12)		(-0,02 to 0,18)		(-0,10 to 0,10)		(-0,03 to 0,16)	
Mostly	205 (29,29)	-0,10		-0,10		-0,06		-0,16**		0,24***	
		(-0,20 to 0,00)		(-0,2 to 0,00)		(-0,16 to 0,04)		(-0,26 to -0,06)		(0,15 to 0,34)	
Definitely yes	174 (24,86)	Reference		Reference		Reference		Reference		Reference	

* *p* < 0.05; ** *p* < 0.01; *** *p* < 0.001

Higher age was significantly associated with higher IES-R scores (B = 0.12), higher level of stress (B = 0.08) and anxiety (B = 0.11). The single status was associated with lower IES-R scores (B = -0.08), but also with lower quality of life (B = -0.10). Compared to people with higher education, significantly higher IES-R results were recorded in people with at most vocational education (B = 0.21). Higher scores in relation to people with higher education in the stress subscale DASS (B = 0.25) were recorded for people with at most vocational education, but lower (B = -0.12) for people with secondary education. A similar situation took place in the anxiety subscale (respectively: B = 0.31; B = -0.19). People with at most vocational education also had a significantly lower quality of life (B = -0.25) in relation to people with higher education.

In relation to the professionally inactive, the professionally active or studying persons were characterized by significantly lower results in the IES-R (respectively: B = -0.10; B = -0.08). Students also experienced lower anxiety (B = -0.11), while working people had a lower level of depression (B = -0.15) than professionally inactive people. In professionally active people, a significantly higher level of quality of life was found (B = 0.10) than in professionally inactive persons. People whose financial resources were not sufficient to satisfy their needs had significantly higher results in IES-R (B = 0.20), higher levels of stress (B = 0.18), anxiety (B = 0.18) and depression (B = 0.20) than persons for whom funds allowed to meet their needs to a large extent. People whose financial resources were totally insufficient or rather insufficient to meet their needs also had a significantly lower quality of life (respectively: B = -0.29; B = -0.28).

### Infectious symptoms, health condition and psychological impact

Table 2 presents the occurrence of disease symptoms, including 2.86% of respondents reporting a fever of 38°C for at least one day during the last two weeks. Some respondents reported a range of physical symptoms, most commonly headache (28.00%), sore throat (16.86%), common cold (16.71%), dizziness (11.00%), cough (10.57%), muscle aches (10.14%), breathing problems (5.43%), and chills (3.00%). Due to the symptoms, 10.14% of respondents consulted a doctor. During the 14 days preceding the study, 1.14% of respondents were hospitalized, 3.71% were tested for COVID-19, and 10.86% were quarantined. 7.00% assessed their health as not very good, 44.29% as good, 37.86% as very good and 10.86% as excellent. 9.29% of respondents had at least 3 chronic diseases, 11.71% - 2 chronic diseases, 25.00% - 1 disease, and 54.00% did not declare the presence of chronic diseases. The vast majority of respondents had medical insurance (92.57%). Linear regression showed that high temperature, headache, sore throat, dizziness, cough, muscle aches, difficulty breathing, chills were significantly associated with higher IES-R, stress, anxiety and depression levels, and lower quality of life. Common cold (coryza) was only associated with a lower quality of life.

**Table 2:** Association between physical health status in the past 14 days and the psychological impact of the 2019 coronavirus disease (COVID-19) outbreak as well as adverse mental health status during the epidemic (*n* = 700)

Variables	***N (%)*** ***Mean (SD)***	Impact of event		Stress		Anxiety		Depression		WHOQOL	
		Beta (95% Confidence Interval) B (95% CI)	R-Squared (R^**2**^)	Beta (95% Confidence Interval) B (95% CI)	R-Squared (R^**2**^)	Beta (95% Confidence Interval) B (95% CI)	R-Squared (R^**2**^)	Beta (95% Confidence Interval) B (95% CI)	R-Squared (R^**2**^)	Beta (95% Confidence Interval) B (95% CI)	R-Squared (R^**2**^)
**Persistent fever (>38 C for at least 1 day)**											
Yes	20 (2,86)	0,18***	0,032	0,20***	0,039	0,21***	0,045	0,22***	0,049	-0,22***	0,050
		(0,11 to 0,25)		(0,12 to 0,27)		(0,14 to 0,29)		(0,15 to 0,29)		(-0,30 to -0,15)	
No	680 (97,14)	Reference		Reference		Reference		Reference		Reference	
**Chills**											
Yes	21 (3,00)	0,11**	0,013	0,14***	0,019	0,13***	0,016	0,20***	0,042	-0,16***	0,025
		(0,04 to 0,19)		(0,06 to 0,21)		(0,05 to 0,20)		(0,13 to 0,28)		(-0,23 to -0,09)	
No	679 (97,00)	Reference		Reference		Reference		Reference		Reference	
**Headache**											
Yes	196 (28,00)	0,21***	0,042	0,26***	0,065	0,20***	0,039	0,21***	0,043	-0,21***	0,044
		(0,13 to 0,28)		(0,18 to 0,33)		(0,12 to 0,27)		(0,14 to 0,28)		(-0,28 to -0,14)	
No	504 (72,00)	Reference		Reference		Reference		Reference		Reference	
**Myalgia**											
Yes	71 (10,14)	0,15***	0,022	0,18***	0,032	0,23***	0,051	0,20***	0,041	-0,22***	0,048
		(0,07 to 0,22)		(0,11 to 0,25)		(0,15 to 0,30)		(0,13 to 0,28)		(-0,29 to -0,15)	
No	629 (89,86)	Reference		Reference		Reference		Reference		Reference	
**Cough**											
Yes	74 (10,57)	0,14***	0,020	0,18***	0,031	0,18***	0,033	0,19***	0,037	-0,21*	0,044
		(0,07 to 0,21)		(0,10 to 0,25)		(0,11 to 0,25)		(0,12 to 0,27)		(-0,28 to -0,14)	
No	626 (89,43)	Reference		Reference		Reference		Reference		Reference	
**Breathing difficulty**											
Yes	38 (5,43)	0,20***	0,041	0,22***	0,047	0,29***	0,086	0,20***	0,042	-0,20***	0,041
		(0,13 to 0,27)		(0,15 to 0,29)		(0,22 to 0,36)		(0,13 to 0,28)		(-0,27 to -0,13)	
No	662 (94,57)	Reference		Reference		Reference		Reference		Reference	
**Dizziness**											
Yes	77 (11,00)	0,22***	0,050	0,28***	0,080	0,38***	0,144	0,26***	0,069	-0,19***	0,036
		(0,15 to 0,30)		(0,21 to 0,35)		(0,31 to 0,45)		(0,19 to 0,33)		(-0,26 to -0,12)	
No	623 (89,00)	Reference		Reference		Reference		Reference		Reference	
**Coryza**											
Yes	117 (16,71)	0,06	0,003	0,07	0,005	0,03	0,001	0,08*	0,006	-0,12**	0,013
		(-0,02 to 0,13)		(0,00 to 0,14)		(-0,04 to 0,11)		(0,01 to 0,15)		(-0,19 to -0,04)	
No	583 (83,29)	Reference		Reference		Reference		Reference		Reference	
**Sore throat**											
Yes	118 (16,86)	0,14***	0,021	0,19***	0,036	0,16***	0,027	0,23***	0,054	-0,22***	0,048
		(0,07 to 0,22)		(0,12 to 0,26)		(0,09 to 0,24)		(0,16 to 0,31)		(-0,29 to -0,15)	
No	582 (83,14)	Reference		Reference		Reference		Reference		Reference	
**Consultation with doctor in the clinic in the past 14 days**											
Yes	71 (10,14)	0,20***	0,040	0,26***	0,068	0,37***	0,138	0,21***	0,045	-0,13***	0,016
		(0,13 to 0,27)		(0,19 to 0,33)		(0,30 to 0,44)		(0,14 to 0,28)		(-0,2 to -0,05)	
No	629 (89,86)	Reference		Reference		Reference		Reference		Reference	
**Recent hospitalization in the past 14 days**											
Yes	8 (1,14)	0,20***	0,042	0,18***	0,033	0,23***	0,053	0,20***	0,041	-0,21***	0,043
		(0,13 to 0,28)		(0,11 to 0,25)		(0,16 to 0,30)		(0,13 to 0,28)		(-0,28 to -0,14)	
No	692 (98,86)	Reference		Reference		Reference		Reference		Reference	
**Recent testing for COVID-19 in the past 14 days**											
Yes	26 (3,71)	0,21***	0,045	0,23***	0,052	0,18***	0,033	0,19***	0,038	-0,25***	0,063
		(0,14 to 0,28)		(0,16 to 0,3)		(0,11 to 0,25)		(0,12 to 0,27)		(-0,32 to -0,18)	
No	674 (96,29)	Reference		Reference		Reference		Reference		Reference	
**Recent quarantine in the past 14 days**											
Yes	76 (10,86)	0,17***	0,027	0,19***	0,038	0,30***	0,088	0,20***	0,042	-0,14***	0,020
		(0,09 to 0,24)		(0,12 to 0,27)		(0,23 to 0,37)		(0,13 to 0,28)		(-0,21 to -0,07)	
No	624 (89,14)	Reference		Reference		Reference		Reference		Reference	
**Current self-rating health status**											
Not very well	49 (7,00)	0,38***	0,138	0,36***	0,160	0,41***	0,183	0,38***	0,138	-0,43***	0,168
		(0,30 to 0,47)		(0,27 to 0,45)		(0,32 to 0,49)		(0,29 to 0,47)		(-0,52 to -0,35)	
Well	310 (44,29)	0,05		0,13**		0,10*		0,06		-0,05	
		(-0,03 to 0,13)		(0,05 to 0,21)		(0,02 to 0,17)		(-0,02 to 0,13)		(-0,12 to 0,03)	
Very well	265 (37,86)	-0,17***		-0,14***		-0,27***		-0,19***		0,19***	
		(-0,25 to -0,09)		(-0,22 to -0,07)		(-0,34 to -0,19)		(-0,26 to -0,11)		(0,12 to 0,27)	
Perfectly	76 (10,86)	Reference		Reference		Reference		Reference		Reference	
**Chronic illness**											
3 or more	65 (9,29)	0,22***	0,048	0,20**	0,034	0,24***	0,041	0,23***	0,040	-0,33***	0,064
		(0,09 to 0,35)		(0,07 to 0,33)		(0,11 to 0,36)		(0,11 to 0,36)		(-0,46 to -0,21)	
2	82 (11,71)	0,05		0,07		0,05		0,03		-0,01	
		(-0,07 to 0,17)		(-0,06 to 0,19)		(-0,07 to 0,17)		(-0,09 to 0,15)		(-0,13 to 0,11)	
1	175 (25,00)	-0,06		-0,10		-0,12		-0,09		0,14	
		(-0,18 to 0,05)		(-0,22 to 0,01)		(-0,23 to 0)		(-0,20 to 0,03)		(-0,02 to 0,25)	
0	378 (54,00)	Reference		Reference		Reference		Reference		Reference	
**Medical insurance coverage** 624 (89,14)											
Yes	648 (92,57)	-0,01	0,000	-0,01	0,000	0,00	0,000	-0,04	0,002	0,08*	0,007
		(-0,09 to 0,06)		(-0,09 to 0,06)		(-0,07 to 0,07)		(-0,12 to 0,03)		(0,01 to 0,16)	
No	52 (7,43)	Reference		Reference		Reference		Reference		Reference	

* *p* < 0.05; ** *p* < 0.01; *** *p* < 0.001

People who consulted a doctor within the last 14 days were hospitalized, tested for COVID-19, were in quarantine, as well as those who assessed their health as not very good and those who had at least 3 chronic diseases had significantly higher IES-R, stress, anxiety and depression level and lower quality of life. People with medical insurance had a significantly higher quality of life than those without insurance.

### Contact history and psychological influence

Table 3 presents the history of contacts with people with confirmed and suspected COVID-19 infection. As many as 9.57% of respondents confirmed close contact with a person infected with COVID-19, 17.43% had indirect contact with an infected person and the same number with a person suspected of being infected with COVID-19 or potentially infected materials. All these persons obtained significantly higher results on the IES-R scale and had significantly higher levels of stress, anxiety and depression, and were characterized by a lower quality of life compared to those who did not have such contact.

**Table 3:** Association between contact history in the past 14 days and the psychological impact of the 2019 coronavirus disease (COVID-19) outbreak as well as adverse mental health status during the epidemic (*n* = 700)

Variables	***N (%)*** ***Mean (SD)***	Impact of event		Stress		Anxiety		Depression		WHOQOL	
		Beta (95% Confidence Interval) B (95% CI)	R-Squared (R^**2**^)	Beta (95% Confidence Interval) B (95% CI)	R-Squared (R^**2**^)	Beta (95% Confidence Interval) B (95% CI)	R-Squared (R^**2**^)	Beta (95% Confidence Interval) B (95% CI)	R-Squared (R^**2**^)	Beta (95% Confidence Interval) B (95% CI)	R-Squared (R^**2**^)
**Close contact with an individual with confirmed infection with COVID-19**											
Yes	67 (9,57)	0,15***	0,024	0,23***	0,051	0,35***	0,121	0,21***	0,045	-0,08*	0,007
		(0,08 to 0,23)		(0,15 to 0,30)		(0,28 to 0,42)		(0,14 to 0,28)		(-0,16 to -0,01)	
No	633 (90,43)	Reference		Reference		Reference		Reference		Reference	
**Indirect contact with an individual with confirmed infection with COVID-19**											
Yes	122 (17,43)	0,15***	0,032	0,21***	0,043	0,28***	0,078	0,19***	0,036	-0,11**	0,013
		(0,08 to 0,23)		(0,14 to 0,28)		(0,21 to 0,35)		(0,12 to 0,26)		(-0,19 to -0,04)	
No	578 (82,57)	Reference		Reference		Reference		Reference		Reference	
**Contact with an individual with suspected COVID-19 or infected materials**											
Yes	122 (17,43)	0,15***	0,022	0,22***	0,050	0,26***	0,070	0,19***	0,037	-0,11**	0,012
		(0,07 to 0,22)		(0,15 to 0,30)		(0,19 to 0,34)		(0,12 to 0,26)		(-0,19 to -0,04)	
No	578 (82,57)	Reference		Reference		Reference		Reference		Reference	

* *p* < 0.05; ** *p* < 0.01; *** *p* < 0.001.

### Information and concerns related to COVID-19 and psychological impact

Table 4 presents the association between knowledge and concerns about the COVID-19 and the psychological impact of the outbreak. The main source of information about the pandemic and the Sars-Cov-2 virus in Poland, for the majority of respondents, was the Internet (62.14%) and television (30.29%). The overwhelming majority of respondents were neutral in relation to the quality of information regarding the pandemic (47.71%) or satisfied (44.71%). Most respondents said that in the initial stage of the pandemic, their probability of contracting COVID-19 was moderate (44.71%). Persons who said that they had a low or very low probability of infection were characterized by significantly lower IES-R scores (B = -0.18; B = -0.18, respectively), lower levels of stress (B = -0.17; B = -0.17, respectively), anxiety (B = -0.19; B = -0.20 respectively), depression (B = -0.13; B = -0.15 respectively) and higher quality of life (B = 0.08; B = 0.16 respectively) than people with a high probability of contact. Most of the Poles surveyed stated that they had a high (45.14%) or moderate (34.00%) chance of surviving an infection with the Sars-Cov-2 virus. People who stated that they had a very low chance of surviving the infection had a significantly higher IES-R score (B = 0.37), significantly higher stress (B = 0.35), anxiety (B = 0.46), depression (0.37) and lower quality of life (B = -0.30). Most of the surveyed respondents had moderate (35.00%), high (32.71%) and very high (12.00%) concerns that their family members might be infected with the Sars-Cov-2 virus. Persons who were very worried had a significantly higher IES-R score (B = 0.29), significantly higher stress (B = 0.26), anxiety (B = 0.22), depression (0.24) and lower quality of life (B = -0.20) than people who were not concerned by this fear. The situation was similar in the case of concerns related to the infection of children.

**Table 4:** Association between knowledge and concerns about the 2019 coronavirus disease (COVID-19) and the psychological impact of outbreak as well as adverse mental health status during the epidemic (n = 700).

Variables	***N (%)*** ***Mean (SD)***	Impact of event		Stress		Anxiety		Depression		WHOQOL	
		Beta (95% Confidence Interval) B (95% CI)	R-Squared (R^**2**^)	Beta (95% Confidence Interval) B (95% CI)	R-Squared (R^**2**^)	Beta (95% Confidence Interval) B (95% CI)	R-Squared (R^**2**^)	Beta (95% Confidence Interval) B (95% CI)	R-Squared (R^**2**^)	Beta (95% Confidence Interval) B (95% CI)	R-Squared (R^**2**^)
**The main source of health information**											
TV	212 (30,29)	0,10*	0,018	0,12*	0,021	0,13**	0,020	0,05	0,020	-0,04	0,009
		(0,00 to 0,20)		(0,02 to 0,21)		(0,04 to 0,23)		(-0,05 to 0,15)		(-0,14 to 0,06)	
Radio	20 (2,86)	-0,05		-0,06		-0,03		-0,06		-0,01	
		(-0,13 to 0,02)		(-0,14 to 0,02)		(-0,11 to 0,05)		(-0,14 to 0,02)		(-0,09 to 0,07)	
Internet	435 (62,14)	0,01		0,01		0,02		0,00		-0,01	
		(-0,09 to 0,10)		(-0,09 to 0,11)		(-0,08 to 0,12)		(-0,10 to 0,10)		(-0,11 to 0,09)	
Family members	23 (3,29)	0,09*		0,098		0,07		0,14***		-0,08*	
		(0,01 to 0,17)		(0,01 to 0,17)		(-0,01 to 0,14)		(0,06 to 0,21)		(-0,16 to -0,01)	
Other sources	10 (1,43)	Reference		Reference		Reference		Reference		Reference	
**Satisfaction with the amount of health information available about COVID-19**											
Satisfied	53 (7,58)	0,14*	0,010	0,13*	0,010	0,11	0,023	0,16**	0,021	-0,18**	0,023
		(0,03 to 0,26)		(0,01 to 0,24)		(0,00 to 0,23)		(0,04 to 0,27)		(-0,29 to -0,06)	
Neither satisfied nor dissatisfied	334 (47,71)	-0,06		-0,04		0,04		-0,01		0,04	
		(-0,18 to 0,05)		(-0,16 to 0,07)		(-0,07 to 0,16)		(-0,13 to 0,10)		(-0,08 to 0,15)	
Dissatisfied	313 (44,71)	Reference		Reference		Reference		Reference		Reference	
**Likelihood of contracting COVID-19 during the current outbreak**											
Very small	52 (7,43)	-0,18***	0,087	-0,17***	0,081	-0,20***	0,103	-0,15***	0,062	0,16***	0,050
		(-0,27 to -0,10)		(-0,25 to -0,09)		(-0,28 to -0,11)		(-0,24 to -0,07)		(0,08 to 0,24)	
Small	161 (23,00)	-0,18***		-0,17***		-0,19***		-0,13**		0,05	
		(-0,26 to -0,11)		(-0,25 to -0,10)		(-0,26 to -0,11)		(-0,2 to -0,05)		(-0,03 to 0,12)	
Moderate	313 (44,71)	-0,05		-0,07		-0,07		-0,09*		0,08*	
		(-0,12 to 0,02)		(-0,14 to 0,01)		(-0,14 to 0,01)		(-0,16 to -0,01)		(0,00 to 0,15)	
High	128 (18,29)	0,08		0,11**		0,18***		0,06		0,03	
		(0,00 to 0,15)		(0,04 to 0,19)		(0,1 to 0,25)		(-0,02 to 0,13)		(-0,05 to 0,11)	
Very high	46 (6,57)	Reference		Reference		Reference		Reference		Reference	
**Likelihood of surviving if infected with COVID-19**											
Very small	8 (1,14)	0,37***	0,069	0,35***	0,037	0,46***	0,067	0,37***	0,055	-0,30**	0,084
		(0,16 to 0,57)		(0,14 to 0,56)		(0,25 to 0,66)		(0,16 to 0,57)		(-0,5 to -0,10)	
Small	34 (4,86)	0,11		0,04		0,05		0,11		-0,10	
		(-0,02 to 0,23)		(-0,09 to 0,17)		(-0,08 to 0,18)		(-0,02 to 0,24)		(-0,22 to 0,03)	
Moderate	238 (34,00)	-0,09		-0,12		-0,13*		-0,14*		0,00	
		(-0,22 to 0,03)		(-0,25 to 0,01)		(-0,26 to -0,01)		(-0,27 to -0,02)		(-0,13 to 0,12)	
High	316 (45,14)	-0,27***		-0,23***		-0,32***		-0,30***		0,35***	
		(-0,39 to -0,14)		(-0,36 to -0,10)		(-0,45 to -0,20)		(-0,43 to -0,17)		(0,23 to 0,48)	
Very high	104 (14,86)	Reference		Reference		Reference		Reference		Reference	
**Concerns about other family members getting COVID-19 infection**											
Very high	84 (12,00)	0,29***	0,085	0,26***	0,071	0,22***	0,050	0,24***	0,050	-0,20***	0,042
		(0,21 to 0,37)		(0,18 to 0,34)		(0,14 to 0,3)		(0,16 to 0,32)		(-0,28 to -0,12)	
High	229 (32,71)	0,07		0,08		0,05		0,03		0,02	
		(0 to 0,15)		(0 to 0,15)		(-0,03 to 0,13)		(-0,05 to 0,11)		(-0,06 to 0,1)	
Moderate	245 (35,00)	-0,08		-0,04		-0,02		-0,04		0,05	
		(-0,16 to 0,01)		(-0,12 to 0,04)		(-0,10 to 0,06)		(-0,12 to 0,04)		(-0,03 to 0,13)	
Small	75 (10,71)	-0,10*		-0,13**		-0,12**		-0,08*		0,09*	
		(-0,18 to -0,02)		(-0,21 to -0,05)		(-0,20 to -0,04)		(-0,16 to 0,00)		(0,01 to 0,17)	
Very small	31 (4,43)	-0,06		-0,05		-0,08		-0,07		0,09	
		(-0,15 to 0,03)		(-0,14 to 0,04)		(-0,17 to 0,02)		(-0,16 to 0,02)		(0,00 to 0,18)	
Not applicable	36 (5,14)	Reference		Reference		Reference		Reference		Reference	
**Concerns about a child younger than 16 years getting COVID-19 infection**											
Very high	43 (6,14)	0,27***	0,044	0,25***	0,036	0,16*	0,035	0,18**	0,018	-0,28***	0,033
		(0,14 to 0,4)		(0,12 to 0,38)		(0,03 to 0,29)		(0,05 to 0,32)		(-0,42 to -0,15)	
High	97 (13,86)	0,09		-0,01		0,01		-0,02		0,03	
		(-0,02 to 0,20)		(-0,12 to 0,10)		(-0,10 to 0,12)		(-0,13 to 0,09)		(-0,08 to 0,14)	
Moderate	251 (35,86)	0,05		0,10*		0,15**		0,07		0,03	
		(-0,05 to 0,16)		(0 to 0,21)		(0,05 to 0,26)		(-0,03 to 0,18)		(-0,07 to 0,14)	
Small	102 (14,57)	-0,18**		-0,16**		-0,17**		-0,10		0,07	
		(-0,29 to -0,07)		(-0,27 to -0,05)		(-0,28 to -0,05)		(-0,21 to 0,01)		(-0,04 to 0,18)	
Very small	41 (5,86)	-0,19**		-0,15*		-0,08		-0,11		0,21**	
		(-0,33 to -0,06)		(-0,28 to -0,01)		(-0,22 to 0,05)		(-0,25 to 0,02)		(0,08 to 0,35)	
Not applicable	166 (23,71)	Reference		Reference		Reference		Reference		Reference	

* *p* < 0.05; ** *p* < 0.01; *** *p* < 0.001.

### Precautions and psychological impact

Table 5 shows the precautions taken by the respondents in the last 14 days, the most frequent being always covering their mouths when coughing and sneezing (61.43%), always avoiding sharing dishes during meals (57.72%), always washing their hands with soap (51.29%), wearing a mask regardless of the presence or absence of symptoms (56.29%), always washing hands after touching contaminated objects (63.86%).

**Table 5:** Association between precautionary measures in the past 14 days and the psychological impact of the 2019 coronavirus disease (COVID-19) outbreak as well as adverse mental health status during the epidemic (n = 700).

Variables	***N (%)*** ***Mean (SD)***	Impact of event		Stress		Anxiety		Depression		WHOQOL	
		Beta (95% Confidence Interval) B (95% CI)	R-Squared (R^**2**^)	Beta (95% Confidence Interval) B (95% CI)	R-Squared (R^**2**^)	Beta (95% Confidence Interval) B (95% CI)	R-Squared (R^**2**^)	Beta (95% Confidence Interval) B (95% CI)	R-Squared (R^**2**^)	Beta (95% Confidence Interval) B (95% CI)	R-Squared (R^**2**^)
**Covering mouth when coughing and sneezing**											
Always	430 (61,43)	-0,13***	0,052	-0,19***	0,040	-0,27***	0,073	-0,22***	0,048	0,20***	0,041
		(-0,2 to -0,06)		(-0,27 to -0,12)		(-0,34 to -0,20)		(-0,29 to -0,15)		(0,13 to 0,28)	
Most of the time	197 (28,14)	0,01		0,06		0,01		0,02		0,00	
		(-0,07 to 0,08)		(-0,02 to 0,13)		(-0,06 to 0,08)		(-0,05 to 0,1)		(-0,07 to 0,07)	
Occasionally at best	73 (10,43)	Reference		Reference		Reference		Reference		Reference	
**Avoiding sharing of utensils during meals**											
Always	404 (57,72)	0,03	0,001	-0,08	0,005	-0,09*	0,007	-0,10***	0,011	0,06	0,003
		(-0,05 to 0,11)		(-0,16 to 0)		(-0,17 to -0,01)		(-0,18 to -0,02)		(-0,02 to 0,14)	
Most of the time	166 (23,71)	-0,01		0,03		0,03		0,00		-0,01	
		(-0,09 to 0,07)		(-0,04 to 0,11)		(-0,05 to 0,11)		(-0,08 to 0,08)		(-0,09 to 0,07)	
Occasionally at best	130 (18,57)	Reference		Reference		Reference		Reference		Reference	
**Washing hands with soap and water**											
Always	359 (51,29)	-0,07***	0,011	-0,13**	0,016	-0,16***	0,022	-0,18***	0,029	0,21***	0,037
		(-0,15 to 0,01)		(-0,21 to -0,05)		(-0,24 to -0,08)		(-0,26 to -0,10)		(0,13 to 0,29)	
Most of the time	306 (43,71)	-0,11		-0,08		-0,08*		-0,11**		0,09*	
		(-0,19 to -0,03)		(-0,16 to 0,00)		(-0,16 to 0,00)		(-0,19 to -0,03)		(0,02 to 0,17)	
Occasionally at best	35 (5,00)	Reference		Reference		Reference		Reference		Reference	
**Washing hands immediately after coughing, rubbing nose, or sneezing**											
Always	132 (18,86)	-0,05	0,009	-0,05	0,002	-0,02	0,006	-0,10	0,006	0,20***	0,025
		(-0,15 to 0,04)		(-0,15 to 0,04)		(-0,12 to 0,07)		(-0,19 to 0,00)		(0,1 to 0,29)	
Most of the time	243 (34,71)	0,12*		0,06		0,09		0,05		-0,08	
		(0,02 to 0,21)		(-0,03 to 0,16)		(-0,01 to 0,19)		(-0,04 to 0,15)		(-0,18 to 0,01)	
Occasionally at best	325 (46,43)	Reference		Reference		Reference		Reference		Reference	
**Wearing mask regardless of the presence or absence of symptoms**											
Always	394 (56,29)	0,03	0,001	-0,04	0,005	0,06	0,009	-0,06	0,007	0,11**	0,011
		(-0,04 to 0,11)		(-0,11 to 0,04)		(-0,02 to 0,13)		(-0,13 to 0,02)		(0,03 to 0,18)	
Most of the time	210 (30,00)	-0,03		-0,05		-0,08*		-0,05		0,00	
		(-0,1 to 0,05)		(-0,13 to 0,02)		(-0,16 to -0,01)		(-0,13 to 0,02)		(-0,07 to 0,08)	
Occasionally at best	96 (13,71)	Reference		Reference		Reference		Reference		Reference	
**Washing hands after touching contaminated objects**											
Always	447 (63,86)	-0,02	0,001	-0,09*	0,011	-0,14***	0,021	-0,15***	0,023	0,11**	0,016
		(-0,10 to 0,05)		(-0,17 to -0,02)		(-0,22 to -0,07)		(-0,22 to -0,07)		(0,04 to 0,19)	
Most of the time	172 (24,57)	0,02		0,06		0,05		0,05		-0,07	
		(-0,06 to 0,09)		(-0,01 to 0,14)		(-0,03 to 0,12)		(-0,02 to 0,13)		(-0,14 to 0)	
Occasionally at best	81 (11,57)	Reference		Reference		Reference		Reference		Reference	
**Feeling that too much unnecessary worry has been made about the COVID-19 outbreak**											
Always	52 (7,43)	0,00	0,000	-0,06	0,003	0,01	0,007	0,00	0,001	-0,01	0,000
		(-0,12 to 0,12)		(-0,18 to 0,06)		(-0,11 to 0,13)		(-0,11 to 0,12)		(-0,12 to 0,11)	
Most of the time	151 (21,57)	0,00		0,01		-0,09		-0,04		0,01	
		(-0,12 to 0,11)		(-0,11 to 0,13)		(-0,21 to 0,03)		(-0,16 to 0,08)		(-0,11 to 0,13)	
Occasionally at best	497 (71,00)	Reference		Reference		Reference		Reference		Reference	
**Average number of hours staying at home per day to avoid COVID-19**											
More than 19 hours	377 (53,86)	0,00	0,001	0,03	0,003	-0,02	0,025	0,05	0,004	-0,04	0,002
		(-0,08 to 0,07)		(-0,05 to 0,1)		(-0,09 to 0,06)		(-0,03 to 0,12)		(-0,11 to 0,04)	
10-19 hours	267 (38,14)	0,04		0,05		0,16***		0,05		0,02	
		(-0,04 to 0,11)		(-0,02 to 0,13)		(0,08 to 0,23)		(-0,02 to 0,13)		(-0,06 to 0,09)	
Up to 9 hours	56 (8,00)	Reference		Reference		Reference		Reference		Reference	

* *p* < 0.05; ** *p* < 0.01; *** *p* < 0.001.

Among them, always washed their hands immediately after coughing, sneezing or rubbing their nose only 18.86%, most of the time (34.71%), unfortunately as many as 46.43% did it only sporadically. Linear regression analysis showed that people who always covered their mouths when coughing or sneezing and always washed their hands with soap and water had significantly lower IES-R scores (B = -0.13; B = -0.07, respectively), lower levels of stress (respectively: B = -0.19; B = -0.13), lower level of anxiety (respectively: B = -0.27; B = -0.16), lower level of depression (respectively: B = -0.22; B = -0.18) and higher quality of life (respectively: B = 0.20; B = 0.21) than people who did it sporadically. People who always avoided sharing utensils had significantly lower levels of anxiety (B = -0.09), lower levels of depression (B = -0.10), and higher quality of life (B = 0.06) compared to those who did so sporadically at most. People who always washed their hands after touching contaminated objects had significantly lower levels of stress (B = -0.09), significantly lower levels of anxiety (B = -0.14), lower levels of depression (B = -0.15) and higher quality of life (B = 0.11) compared to people who did it sporadically.

## Discussion

Our findings indicate the occurrence of significant psychological reactions of the Polish adult population, in general, in the first period of the COVID-19 pandemic. The psychological impact of the COVID-19 outbreak in Poland, measured using the IES-R scale, showed an average post-traumatic stress at the level of 29.75 (SD = 16.05). Out of all respondents, 38.71% reported a minimal psychological impact, 20.71% indicated a mild psychological impact, and a moderate and strong one occurred in 40.58%. A similar average level of suffering from traumatic events related to the outbreak of the pandemic occurred in China, with other countries that measured such it this, a lower level of post-traumatic stress has occurred. Wang et al., examining 1,210 people representing an average population in China using an online survey, found a slightly higher psychological impact of the pandemic measured with the IES-R scale (mean score 32.98, SD = 15.42). In China, a slightly smaller proportion of respondents reported a slight psychological impact of the pandemic (24.5%), a similar proportion had a mild impact (21.7%), and a slightly greater proportion had a moderate and severe impact (53.8% in total) [25]. Di Giuseppe et al. studied 5,683 Italians in the early period of the pandemic using the IES-R scale. The average IES-R value was slightly lower than in Poland and China and amounted to 24.72 (SD = 16.10) [36]. Hao et al. conducted an online survey of people with and without mental illness during the COVID-19 pandemic. They found statistically significantly higher levels of posttraumatic stress measured IES-R in people with mental disorders (mean IES-R = 17.7; SD = 14.2) than in people without such disorders (mean IES-R = 11.3; SD=10.1) [37]. Chew et al. studied 21,906 health care workers from major hospitals in Singapore and India. The authors found the average level of IES-R in the studied group at a lower level (8.29; SD 9.79) than in the studied population representing the general population in Poland, and this effect was also found in a lower percentage of respondents (7.4%) [38]. Tee et al. analyzed 2,037 questionnaires collected through an online survey among the Luzon Islands of the Philippine community. The psychological impact of the COVID-19 pandemic measured with the IES-R showed a sample mean score of 19.57 (SD = 13.12) [39].

In Poland, mild depression was found in 14.71%, moderate in 22.00%, and severe and extremely severe in 11.34% of respondents. In their studies, Wang et al. found occurrence of depression in a smaller percentage of respondents, including mild depression in 13.8%, moderate depression in 12.2%, and severe and extremely severe depression in 4.3% [25]. In the Philippines, depression was found even less frequently, 11.9% reported mild depressive symptoms, 12.7% - moderate, and 4.2% - reported severe to extremely severe depression [39]. Hao et al. found a statistically significantly higher level of depression in patients with mental illness than in patients without diagnosed mental illness. The average level of depression in both groups was lower than in the average sample of the Polish population [37]. García-Álvarez et al. in the studied sample of Spaniards found mild depression in 28.0% of the respondents, moderate depression in 12.5%, and severe and extremely severe depression in 6.2% [40]. In the studied group in Poland, a mild level of anxiety was found in 7.14%, in 20.86% - moderate, and in 11.24% - a strong and extremely strong anxiety. In the study group in China, mild anxiety level was found in 7.5%, moderate - in 20.4%. Slightly fewer respondents in China (8.4%) than in Poland suffered from severe and extremely severe anxiety [25]. In the Philippines, 9.6% of respondents reported mild anxiety symptoms, 17.7% - moderate anxiety symptoms, and 11.1% - symptoms of severe to extremely severe anxiety [39]. The situation related to experiencing anxiety in the Philippines was similar to the situation in Poland.

In the studied group of Poles, mild stress was present in 37.29% of the respondents, moderate - in 11.86%, and severe stress in 25.71%. In the group of Chinese, the presence of stress was found in a smaller percentage of the respondents, in 24.1% mild stress was found, in 5.5% - moderate, and in 2.6% - severe and extremely severe stress [25]. Tee et al. found mild stress signals in 26.4% of Filipinos, moderate in 9.5%, and at least severe stress in 3.9% [39]. In general, it can be stated that the percentage of people experiencing stress in the Philippines was slightly lower than in Poland.

The average quality of life level in Poland was the lowest for the physical domain and it amounted to 49.56 (SD=11.71). The highest average standard of living was in the environmental domain and it was 65.83 (SD=15.12) and in the social domain and it was 65.82 (SD=19.41). The standard of living in the psychological domain was 60.26 (SD=13.14). The situation was slightly different for Cawla et al., who studied the population of medical students in India during the pandemic, and who found the mean WHOOL-BREF domains scores highest for the environmental domain (72.10 ± 13.0), then the physical domain (67.23 ± 13.74), social (57.13 ± 20.1), and the lowest for the psychological domain (52.10 ± 17.45) [41].

In Poland, significantly higher results of the IES-R scale during the first wave of the pandemic were found in older people, living in a relationship, with a lower level of education, with children aged 16 and over, and with insufficient or small financial resources to meet their needs. It was different in China, where women and students had higher IES-R scores [25]. In the Philippines, significantly higher psychological impact in the first period of the pandemic was found in young people and students, as well as in women, single people, people with lower education and non-health care workers [39]. In Italy, the highest level of post-traumatic stress was recorded in women and middle-aged people (30-49 years) [36]. In Poland, in the first period of the pandemic, a significantly higher level of stress was found in older people, with a lower level of education, with children aged 16 and over, and with insufficient or small financial resources to meet their needs. In China, a significantly higher level of stress was found in men [25]. In the Philippines, significantly higher levels of stress were found in women, young people, single people, people with lower education, students and non-health professionals [39].

In Poland, a significantly higher level of anxiety was characteristic of the female sex, the elderly, people with at most vocational education, with children aged 16+ and insufficient or small financial resources to meet their needs. Similarly, in the Chinese study, men had a significantly higher level of anxiety than women [25]. In the Philippines, significantly higher levels of anxiety were observed in women, younger people and students, single people, people with lower education and parents [39]. Among the surveyed Poles, a significantly higher level of depression was observed in people with at most vocational education, professionally inactive, with children aged 16 and over, and with insufficient or small financial resources to meet their needs. In China, a significantly higher level of depression was found in men and students [25]. In the Philippines, significantly higher levels of anxiety were found in younger people and students, single people, people with lower education, non-health professionals and parents [39]. In Poland, single people, with lower education and low income had a significantly lower quality of life. Gender had no effect on quality of life. In India, male medical students had a higher quality of life than female students [41]. In Italy, healthcare workers who had unprotected exposure to COVID-19 patients had an increase in physical and psychological symptoms, even without developing disease, while infected workers had a sharp increase in anxiety, depression, and sleep disturbances [42]. The prolonged stress associated with the pandemic increases the incidence of burnout syndrome in health care workers, characterized by emotional exhaustion, depersonalization, and low personal accomplishment [43, 44].

The six most common infectious symptoms of the studied group of Poles in the first weeks of the pandemic occurred in the following order: headache, sore throat, coryza, dizziness, cough and muscle pain. In China, these were also the most common symptoms [25]. In the study group in Poland, linear regression showed that high temperature, headache, sore throat, dizziness, cough, muscle aches, difficulty breathing, and chills were significantly associated with higher IES-R, stress, anxiety and depression, and lower levels of quality of life. The Chinese analogous linear regression similarly showed that: chills, muscle aches, cough, dizziness, coryza and sore throat were significantly associated with higher IES-R scores, higher levels of stress, anxiety, and depression [25]. The same was true in the Philippines [39]. Linear regression conducted by Chew et al. revealed that the presence of physical symptoms in health care workers was associated with higher mean scores on the IES-R and on all subscales of the DASS-21. This relationship was especially present in three respiratory symptoms (sore throat, dyspnea and cough) [38]. Hao et al. found similar results [37].

In Poland, due to the infectious symptoms, 10.14% of respondents consulted a doctor. During the 14 days preceding the study, 1.14% of respondents were hospitalized, 3.71% were tested for COVID-19, and 10.86% were quarantined. In the Chinese study group, a significantly smaller percentage of respondents consulted a doctor (3.5%) in the two weeks preceding the study, were admitted to the hospital (0.3%), were tested for COVID-19 (0.9%) and were quarantined (2.1%) [23]. In Poland, the majority of respondents assessed their health as good (44.29%) or very good (37.86%). Similarly, in China, 68.3% reported good or very good health. However, in Poland (54.00%), significantly fewer people than in China (93.6%), reported that they did not suffer from any chronic disease [25]. In Poland, people who consulted a doctor during the last 14 days, were hospitalized, tested for COVID-19, or were in quarantine, had significantly higher IES-R, stress, anxiety and depression level, and a lower level of quality of life. The same was true in China [25] and the Philippines [39]. In Poland, people who assessed their health worse and suffered from chronic diseases had a significantly higher level of IES-R, stress, anxiety and depression. The same was true in China [25] and the Philippines [39].

In Poland, people who confirmed close or indirect contact with a person infected with COVID-19, or suspected of such infection, or contact with materials potentially infected with COVID-19 had significantly higher IES-R scores and a significantly higher level of stress, anxiety and depression as well as a lower quality of life in relation to people who did not have such contact. In China, no such intensification of emotional symptoms was recorded in similar situations [25].

The main source of information about the pandemic in Poland for the majority of respondents was the Internet (62.14%) and television (30.29%). In China and the Philippines, the Internet was by far the most common source of information about the pandemic [25, 39].

Most respondents in Poland stated that in the initial stage of the pandemic, the probability of contracting the Sars-Cov-2 virus for them and their families was moderate, high or very high. These people had significantly higher IES-R scores and higher levels of stress, anxiety and depression, as well as a lower quality of life. The same was true for the respondents in China and the Philippines [25, 39]. In people who claimed that they had little chance of surviving this infection, psychological indicators were significantly higher, and the quality of life was significantly lower. It was similar in other countries [25, 39].

Most of the surveyed Poles always covered their mouths when coughing and sneezing, avoided sharing dishes with meals, washed their hands with soap, wore a mask regardless of the presence or absence of symptoms, and washed their hands after touching potentially contaminated objects. Unfortunately, always washed their hands immediately after coughing, sneezing or rubbing their nose, about one in five people only. Linear regression analysis showed that Poles who always adhered to the rules of hygiene had a significantly lower level of emotional symptoms compared to people who did not. Interestingly, no such relationship has been reported for wearing masks. Similar relationships were reported in China, except that wearing masks was associated with lower psychological impact scores. The rarity of wearing masks, regardless of the presence or absence of symptoms, was significantly associated with higher IES-R scores [25]. In Poland, people taking precautions against COVID19 infection had a significantly higher quality of life.

### Limitations

The limitation of the research is the cross-sectional measurement of the study, which excludes longitudinal observation of changes. Further studies should also be carried out in a larger group of respondents.

## Conclusion

The pandemic is having a significant impact on human mental health. In Poland, this impact appears to be higher than in other countries that have made such an assessment. Relatively high average level of post-traumatic stress, stress, anxiety and depression as well as low quality of life make it necessary to consider interventions that will favor the use of more adaptive defense mechanisms and build mental resilience during an infectious disease pandemic and its long-term consequences. Our research made it possible to identify groups of special risk to which psychological support should be addressed in the first place.

In a pandemic and changing world situation, psychological support services are urgently needed and should be based on coping strategies. The intervention plan should provide broad access to appropriate information, training and responses to life-threatening situations, and psychological support in coping with anxiety and stress, especially in groups most exposed to psychological stress related to potentially life-threatening situations. The use of psychologists' cooperation with schools and universities in this area and the introduction of mental health monitoring during periodic medical examinations performed by occupational medicine doctors should be considered. These activities should take into account the principles of military psychology in crisis situations [45, 46]. In addition, consideration should be given to using various spiritual care programs and other approaches to reduce anxiety, stress and emotional exhaustion, especially in the most vulnerable groups [47, 48].

## Data Availability

The dataset used and analyzed during this study is available from the Rzeszów University Repository: http://repozytorium.ur.edu.pl/handle/item/6199
